# Analysis of NCL Proteins from an Evolutionary Standpoint

**DOI:** 10.2174/138920208784139573

**Published:** 2008-04

**Authors:** Neda E Muzaffar, David A Pearce

**Affiliations:** 1 Center for Neural Development and Disease, University of Rochester School of Medicine and Dentistry, Rochester, NY 14642, USA; 2 Aab Institute of Biomedical Sciences, Department of Biochemistry and Biophysics, University of Rochester School of Medicine and Dentistry, Rochester, NY 14642, USA; 3 Department of Neurology, University of Rochester School of Medicine and Dentistry, Rochester, NY 14642, USA

## Abstract

The Neuronal Ceroid Lipofuscinoses (NCLs) are the most common group of neurodegenerative disorders of childhood. While mutations in eight different genes have been shown to be responsible for these clinically distinct types of NCL, the NCLs share many clinical and pathological similarities. We have conducted an exhaustive Basic Local Alignment Search Tool (BLAST) analysis of the human protein sequences for each of the eight known NCL proteins- CLN1, CLN2, CLN3, CLN5, CLN6, CLN7, CLN8 and CLN10. The number of homologous species per CLN-protein identified by BLAST searches varies depending on the parameters set for the BLAST search. For example, a lower threshold is able to pull up more homologous sequences whereas a higher threshold decreases this number. Nevertheless, the clade confines are consistent despite this variation in BLAST searching parameters. Further phylogenetic analyses on the appearance of NCL proteins through evolution reveals a different time line for the appearance of the CLN-proteins. Moreover, divergence of each protein shows a different pattern, providing important clues on the evolving role of these proteins. We present and review in-depth bioinformatic analysis of the NCL proteins and classify the CLN-proteins into families based on their structures and evolutionary relationships, respectively. Based on these analyses, we have grouped the CLN-proteins into common clades indicating a common evolving pathway within the evolutionary tree of life. CLN2 is grouped in Eubacteria, CLN1 and CLN10 in Viridiplantae, CLN3 in Fungi/ Metazoa, CLN7 in Bilateria and CLN5, CLN6 and CLN8 in Euteleostomi.

## INTRODUCTION

The neuronal ceroid lipofuscinoses (NCLs, also known as Batten disease) are the most common neurodegenerative disease occurring in childhood with an overall frequency of approximately 1:12,500 [1]. They are a group of neurodegenerative storage disorders inherited in an autosomal recessive manner and are characterized by the ubiquitous accumulation of an autofluorescent lipopigment in tissues of the affected individual [2]. Clinical features are characterized by impaired vision, progressive neurodegeneration resulting in varying degrees of seizures, ataxia progressive psychomotor deterioration and eventually premature death.

The different types of NCLs, which have been categorized on the basis of the age of onset of disease, are infantile NCL (INCL), late-infantile NCL (LINCL), juvenile NCL (JNCL or Battens) and adult NCL (ANCL or Kufs disease). Other variant forms are Finnish variant late-infantile NCL, variant late-infantile NCL, Turkish variant late-infantile NCL, Northern epilepsy (EPMR) and Congenital NCL (CNCL). These NCLs result from mutations in the CLN genetic loci *CLN1, CLN2, CLN3, CLN4, CLN5, CLN6, CLN7*, *CLN8* and *CLN10*, respectively [3]. 

To date, the only NCL proteins whose function has been elucidated are palmitoyl protein thioesterase (PPT1) encoded by *CLN1*; tripeptidyl peptidase 1 (TPP1) by *CLN2*; and cathepsin D by *CLN10*. CLN3, CLN5, CLN6, CLN7, and CLN8 while having been identified, have no definitive functional classification. The genes for *CLN4* and *CLN9* remain to be identified.

## CLN1/PPT1 (INCL)

### Disease Progression

Infantile NCL is the most severe form of NCL and accounts for 25% of all cases in the US [4]. Children with INCL start to show symptoms at around 6-12 months of age when signs of decreased head growth and psychomotor degeneration become apparent. They suffer from progressive vision loss which results in complete blindness by the age of 2. Within the following year most of the affected infants begin to lose higher brain functions and survive into their early to mid teenage years [5-7].

### Disease Autofluorescent Storage Material 

All tissues in the body show cellular accumulation of autofluorescent storage material characterized by a mix of saposins A and D. The saposin proteins are involved in the degradation of sphingolipids in lysosomes [8, 9].

### *CLN1* or *PPT1*Gene and PPT1 Protein

INCL results from mutations in the *CLN1* gene, localized to human chromosome 1p32 [10] which encodes the enzyme palmitoyl protein thioesterase 1 (PPT1) [11]. PPT1 is a soluble lipase that cleaves fatty acids from cysteine residues in proteins during lysosomal protein degradation [12-14]. Mutations in *CLN1* cause decreased enzyme activity that can be readily detected diagnostically [15-19]. There is a good correlation between the level of measurable enzyme activity and the severity of clinical phenotype [20].

### PPT1 Structure

The 37kD, 306 amino acid enzyme contains motifs characteristic of other thioesterases i.e., a G-X-S-X-G in the N-terminal half of the protein and a G-D-H near the C-terminus [11]. X-ray crystallographic studies of the structure of PPT1 have provided insight into the molecular basis for phenotypes of INCL associated with known PPT1 mutations [21]. PPT1 is a globular protein with a classical alpha/beta hydrolase fold typical of lipases. There is a hydrophobic groove in the center of the enzyme which binds to the palmitate portion of the substrate that is to be depalmitoylated. The classical hydrolytic triad consists of serine(S)-115, aspartate (D)-233 and histidine (H)-289. As a result, the mutations that will affect the catalytic activity of PPT1 most severely are the ones that affect residues near the active site and in the hydrophobic core of the enzyme because they affect the folding of the enzyme. Therefore, these mutations correlate with the most severe phenotypes seen in INCL patients. Other mutations which affect the peptide binding pocket or the periphery of the enzyme will still allow residual enzyme activity and have been observed to result in the less severe, late-onset phenotype of INCLs [22]. Over 40 mutations have been described in *CLN1* including missense, nonsense, small deletions or insertions, and mutations affecting splice sites [23]. Mutations leading to decreased enzyme stability and inactivity cause infantile-onset INCL [20].

### PPT1 Function

Palmitoylation is a lipid-modification which attaches a 16-carbon fatty acid chain *via* a labile thioester linkage to cysteine residues. The palmitoylated proteins eventually undergo depalmitoylation in the course of their degradation and disposal *via* the lysosome. PPT1, which is a soluble lipase, is involved in the depalmitoylation of these palmitoylated proteins and is targeted to the lysosomes to execute this function. The targeting of PPT1 to the lysosome occurs through a phosphate modification on mannose residues of asparagines-linked oligosaccharides *via* binding to the mannose 6-phosphate receptor [13, 24]. PPT1 was first purified from bovine brain (30,000-fold enrichment) on the basis of its ability to cleave palmitate from a model^3^H-palmitoylated substrate, H-Ras [12, 25]. In addition to this substrate, a number of other *S*-acylated proteins and peptides are substrates, including palmitoylated G_α _ proteins [25] and fatty acyl-CoAs.

### Evolutionary Relationships- Alignment, Conserved Domains and Mutations

The highest incidence of occurrence of INCL is in Finland where the carrier frequency estimated is 1:70 [11]. Over 90% of these Finnish patients are homozygous for a severe missense point mutation (R122W) leading to enzyme inactivity [23]. Other *CLN1* mutations that result in the severe disease phenotype are H39Q, G42E and V181M. However, mutations such as T75P, G250V, D79G and Q177E result in a milder phenotype with later ages of onset of disease [17, 26, 27].

**Fig. (1) Conserved Domains of human PPT1.**
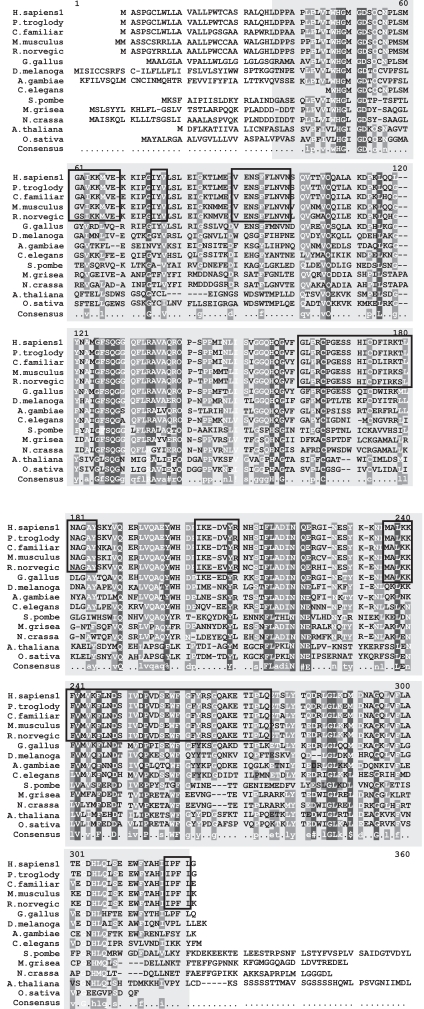
The conserved domain of human PPT1 is Palmitoyl protein thioesterase (pfam02089), indicated by the box shaded in grey that spans the multiple sequence alignment (MSA). It is 279 amino acids in length and aligns 99.6% with the human PPT1 protein from residue 28 to residue 305. The MSA generated by MultAlin highlights individual residues with high consensus value (above 90%) in black and those with low consensus value (above 50%) in gray. All other neutral residues are not highlighted. There are regions of high sequence homology that are not indicated by Multalin; these are shown by black boxes around the conserved regions.

**Fig. (2) Phylogenetic tree of PPT1 homologs.**
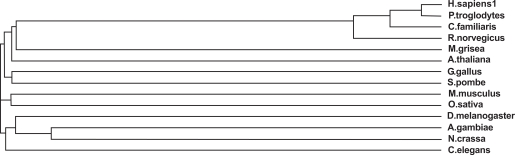
The phylogram tree is generated using the program ClustalW. In the above figure the branch lengths of the 14 homologous sequences are proportional to the amount of inferred evolutionary change.

The severity of these disease causing point mutations can be correlated to their degree of conservation in a multiple sequence alignment of all the known homologs of PPT1 (Fig. **1**). HomoloGene detected 14 homologs for PPT1 which are present in *H. sapiens, P. troglodytes, C. familiaris, M. musculus, R. norvegicus, G. gallus, D. melanogaster, A. gambiae, C. elegans, S. pombe, M. grisea, N. crassa, A. thaliana* and * O. sativa* (Fig. **2**). The domain that is conserved in all of these sequences homologous to PPT1 is 279 amino acids in length which spans almost the entire length of the 306 amino acid PPT1 in *H. sapiens * (Fig. **1**). The multiple sequence alignment was generated using MultAlin which labels conserved mutations in black and grey. It was observed that the early onset associated severe mutations such as R122W, H39Q and V181M were highly conserved in all the homologs of PPT1. 

## CLN2/TPP1 (LINCL)

### Disease Progression

Late Infantile NCL (LINCL) results from mutations in the *CLN2* gene. The symptoms manifest in children at 2 to 4 years of age with progressive neurodegeneration, complete loss of motor function, vision and speech eventually resulting in premature death by the ages 8 to 14. LINCL is associated with instances of generalized gangliosidosis, severe neuronal loss and gliosis of brain tissue and widespread accumulation of storage material in lysosomes [28-33].

### Disease Autofluorescent Storage Material

The principal protein component of the storage bodies observed in patients with LINCL is the subunit c of the mitochondrial ATP synthase [34, 35].

### *CLN2* or *TPP1* Gene and TPP1 Protein

The *CLN2* gene contains 13 exons spanning a 6.65 kb region on chromosome 11p15 [36]. The gene was mapped by means of a proteomics approach that compared brain lysosomal protein contents of normal controls to patients afflicted with LINCL [36].

*CLN2* encodes a lysosomal serine protease [37], tripeptidyl-peptidase I (TPP I) [38, 39], which is 563 amino acids in length and 46 kD in size [40]. TPP1 is a member of the family of serine carboxyl proteinases which play a crucial role in lysosomal protein degradation. 

### TPP1 Structure

Human TPP1 cDNA encodes a preproenzyme of 563 amino acid residues, which includes a 19 amino acid signal peptide cleaved off cotranslationally, a 176 amino acid prodomain removed during the maturation process, and a mature enzyme of 368 amino acid residues [37, 40-42].

### TPP1 Function

TPP1 functions by sequentially removing tripeptides from the N-termini termini of small polypeptides that are sent to the lysosome to be degraded [43-45]. In addition, it has a minor endoprotease activity [46]. When the aminopeptidase activity of TPP1 is disrupted as a result of several mutations [47] it results in the late infantile variant of NCL (LINCL). Subunit c of mitochondrial ATP synthase, the major protein component of the storage material, is likely to be a substrate of CLN2 [48].

### Evolutionary Relationships- Alignment, Conserved Domains and Mutations

**Fig. (3) Conserved Domains of human TPP1.**
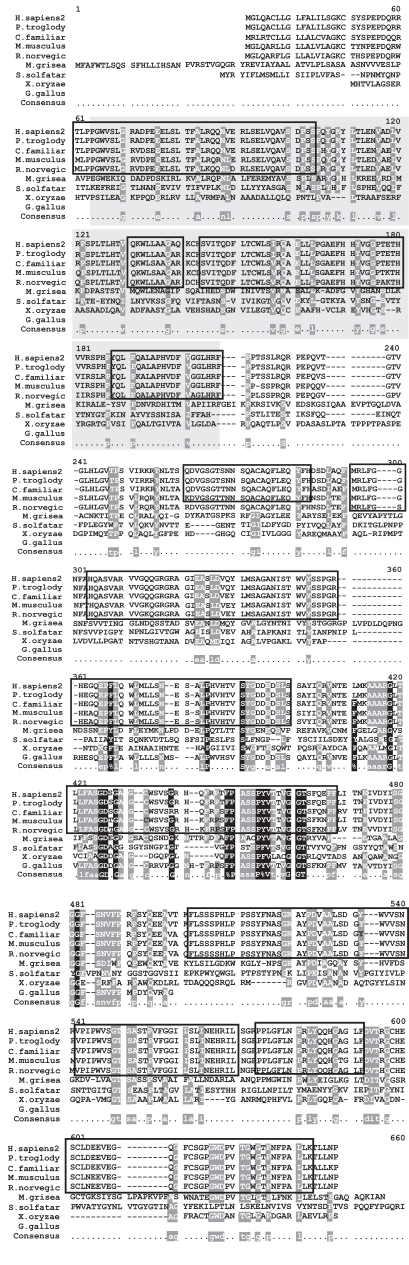
TPP1 in humans contains the conserved Pro-kumamolisin activation domain (pfam09286), indicated by the box shaded in grey. Members of this family are found in various subtilase propeptides, and adopt a ferredoxin-like fold, with an alpha+beta sandwich. Cleavage of the domain results in activation of the peptide. The conserved domain sequence is 142 amino acids in length and aligns 100% with TPP1 protein sequence. The MSA generated by MultAlin highlights individual residues with high consensus value (above 90%) in black and those with low consensus value (above 50%) in gray. All other neutral residues are not highlighted. There are regions of high sequence homology that are not indicated by Multalin; these are shown by black boxes around the conserved regions.

Mutational analyses, inhibition studies and sequence similarities with other family members have identified Ser280, Glu77, and Asp81 as the catalytic residues in TPP1 [49]. These three residues are highly conserved in the multiple sequence alignment (Fig. **3**) of the 9 homologous TPP1 proteins present in *H. sapiens, P. troglodytes, C. familiaris, M. musculus, R. norvegicus, G. gallus, M. grisea, X. oryzae * and * S. solfataricus*.

**Fig. (4) Phylogenetic tree of TPP1 homologs.**
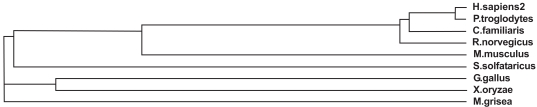
The phylogram tree is generated using the program ClustalW. In the above figure the branch lengths of the 9 homologous sequences are proportional to the amount of inferred evolutionary change.

To date, 52 *CLN2* mutations have been associated with LINCL [47] but the most widespread mutation is the R208X mutation which results in the premature termination of TPP1 synthesis resulting in no translational product [32, 50-52]. As with the essential catalytic residues, this residue is also highly conserved among the homologous species (Fig. **4**) as it has a critical role in the proper functioning of TPP1. The missense mutations that are found in patients with LINCL- N286S, I287N, T353P and Q422H result in amino acid sub-stitutions that induce major misfolding of the precursor peptidase. Consequently, post-translational processing and lysosomal targeting of tripeptidyl-peptidase I is affected. These amino acids, as would be expected, are also present in regions of the protein that are conserved highly by evolution as they are essential for the proper functioning of TPP1 [32]. 

## CLN3 (JNCL)

### Disease Progression

JNCL is the most common of the neurodegenerative disorders and is characterized clinically by vision loss at 3- 5 years of age, seizures of increasing severity followed by progressive psychomotor decline resulting in premature death in the late 20s to early 30s. Though this represents the typical disease course, the onset and severity of disease symptoms can vary depending on the causative genetic mutation as well as environmental influences [53, 54].

### Disease Autofluorescent Storage Material

The AFSM accumulates in the lysosomes of tissues of the patients suffering from Batten disease. The main component of the storage material in these patients is the subunit c of the F_o _region of the ATP synthase complex of the inner mitochondrial membrane [55]. 

### *CLN3* Gene and CLN3 Protein

The *CLN3* gene contains 15 exons and spans a 15 kb region between chromosome 16p12.1- p11.2. The *CLN3* gene encodes the hydrophobic CLN3 protein which is 438 amino acids in length [56, 57].

### CLN3 Structure

The CLN3 protein is predicted to contain 6 transmembrane domains [58] and has been shown to reside in the lysosomal and endosomal membrane of cells [59-61].

### CLN3 Function

CLN3 has been implicated in controlling the acidic pH in lysosomes. Studies of fibroblasts from Batten disease patients show elevated lysosomal pH [62] and yeast lacking the CLN3 protein homolog, Btn1p, have abnormal vacuolar pH in the early phases of growth. This defect in Btn1p lacking yeast strains can be reversed by complementation with either the yeast wild-type *btn1* gene or human *CLN3* [63, 64].

In addition to its role in regulating lysosomal pH, CLN3 has also been suggested to function in maintenance of biophysical membrane properties [65], control of apoptosis [66, 67] and control of protein trafficking [68]. However, the role of CLN3 in the pathological mechanism leading up to Batten disease still remains unclear.

### Evolutionary Relationships- Alignment, Conserved Domains and Mutations

**Fig. (5) Conserved Domains of human CLN3.**
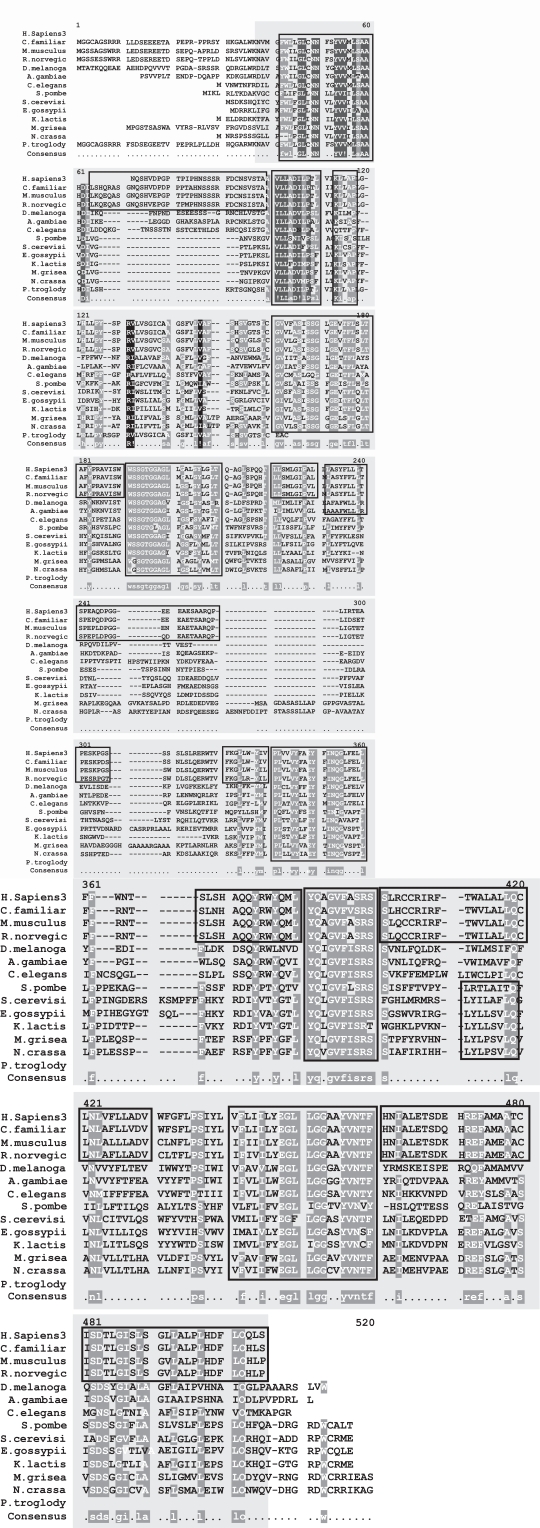
The conserved domain is the human CLN3 protein indicated by the box in grey. A missense mutation of glutamic acid (E) to lysine (K) at position 295 in the human protein has been implicated in Juvenile neuronal ceroid lipofuscinosis (Batten disease). It is 409 amino acids in length and aligns 92% with the human CLN3 protein from residue 4 to residue 368. The MSA generated by MultAlin highlights individual residues with high consensus value (above 90%) in black and those with low consensus value (above 50%) in gray. All other neutral residues are not highlighted. There are regions of high sequence homology that are not indicated by Multalin; these are shown by black boxes around the conserved regions.

**Fig. (6) Phylogenetic tree of CLN3 homologs.**
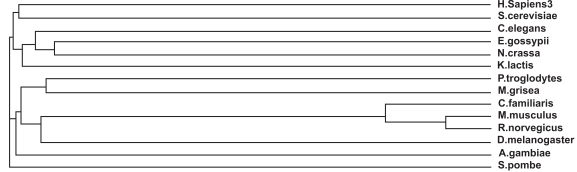
The phylogram tree is generated using the program ClustalW. In the above figure the branch lengths of the 14 homologous sequences are proportional to the amount of inferred evolutionary change.

The mutation responsible for 85% of Batten disease chromosomes is a 1.02 kb deletion resulting in a frameshift mutation that generates a premature termination codon. The result of this mutation is a truncated protein 181 amino acids in length, consisting of the first 153 residues of the protein, followed by 28 novel amino acids before the stop codon [69]. Patients who inherit this mutation homozygously always manifest severe symptoms including blindness, epilepsy, dementia and premature death at approximately 24 years of age. Additionally, 31 other Batten disease mutations have been described [70] which can result in JNCL when inherited in a compound heterozygous manner. Several of these mutations described to result in JNCL cause a disruption or deletion of the highly conserved stretches of amino acids in CLN3, 184**WSSGTGGAGLLG**195, 291**VYFAE**295 and 330**VFASRSSL**337 [67]. These regions are highly conserved (Figs. **5** and **6**) in the CLN3 homologs- *H. sapiens, P. troglodytes, C. familiaris, M. musculus, R. norvegicus, C. elegans, D. melanogaster, A. gambiae, N. crassa, M. grisea, S. pombe, K. lactis, S. cerevisiae* and * E. gossypii*.

## CLN5 (FINNISH VARIANT LINCL)

### Disease Progression

The Finnish variant of late-infantile NCL (Finnish vLINCL or variant Jansky-Bielschowsky disease) is described in Finnish patients with onset at 2 to 7 years of age due to mutations in the*CLN5* gene. The children are afflicted with problems in concentration, motor coordination, mental retardation, visual failure, ataxia, myoclonus and epilepsy [71]. The age at death varies considerably from 14 to 36 years [72-74].

### Disease Autofluorescent Storage Material

Finnish variant LINCL, like the classical LINCL and juvenile variants, contains storage material whose main component is the subunit c of hydrophobic protein mitochondrial ATP synthase. The storage material accumulates in the lysosomes of cells [75] of the patients afflicted by Finnish LINCL.

### *CLN5* Gene and CLN5 Protein

Finnish vLINCL is caused by defects in the CLN5 gene present on chromosome 13q22 [76]. It consists of four exons that span 13kb of genomic DNA and encode a 407 amino acid protein, CLN5, which is 60 kD in size.

### CLN5 Structure

The CLN5 protein is predicted to be a transmembrane protein [77]. However, co-immunoprecipitation experiments by Isosomppi *et al*. (2002) [78] have suggested that CLN5 may represent a soluble lysosomal glycoprotein which is glycosylated and targeted to lysosomes. Soluble and membrane bound forms of the CLN5 protein may exist due to the use of alternative initiation methionines [78, 79].

### CLN5 Function

The function of CLN5 is currently unknown.

### Evolutionary Relationships- Alignment, Conserved Domains and Mutations

**Fig. (7) Conserved Domains of human CLN5.**
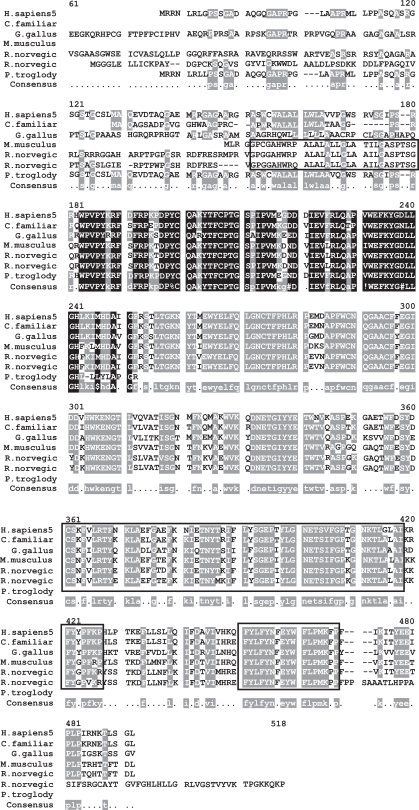
There are no known conserved domains in the human CLN5 protein. However, the MSA of CLN5 and its homologs show regions of high sequence similarity that may indicate potential unidentified domains. The MSA generated by MultAlin highlights individual residues with high consensus value (above 90%) in black and those with low consensus value (above 50%) in grey. All other neutral residues are not highlighted. There are regions of high sequence homology that are not indicated by Multalin; these are shown by black boxes around the conserved regions.

**Fig. (8) Phylogenetic tree of CLN5 homologs.**
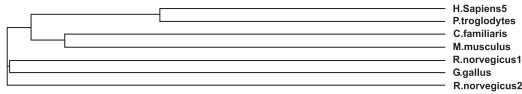
The phylogram tree is generated using the program ClustalW. In the above figure the branch lengths of the 7 homologous sequences are proportional to the amount of inferred evolutionary change.

To date, four disease mutations have been described in CLN5 [74, 77] of which three result in premature termination of the polypeptide chain. The most common mutation among Finnish CLN5 patients is a 2 bp deletion resulting in Tyr392Stop. The other two mutations resulting in truncated polypeptides are G1517A and the SWE mutation INS(C) 1961. The fourth CLN5 mutation, G2127A results in an amino acid substitution of Asp279Asn [78]. All of these sites are highly conserved throughout evolution in C. familiaris, M. musculus, R. norvegicus and P. troglodytes (Figs. **7** and **8**).

## CLN6 (VARIANT LINCL)

### Disease Progression

Variant LINCL patients display the same symptoms of the disease as classical LINCL patients but the onset is delayed and the course is milder [80].

### Disease Autofluorescent Storage Material

Variant LINCL AFSM material is largely composed of storage bodies in the lysosome whose primary protein content is the subunit c of the mitochondrial ATP synthase [81].

### CLN6 Gene and CLN6 Protein

*CLN6* present on chromosome 15q21-23 [76] causes variant late-infantile disease (vLINCL) in families of Indian ancestry [36] and in descendants of Spanish settlers in Costa Rica [82]. The *CLN6* gene contains 7 exons that span approximately 23 kb of genomic DNA. A single 2.4kb mRNA is predicted to encode a 311 amino acid that is 30 kD in size, resides in the endoplasmic reticulum (ER), and has unknown function [83-86].

### CLN6 Structure

CLN6 is predicted to have 7 transmembrane domains [84] and is predicted to be an ER resident membrane protein [85, 86] . It contains an N-terminal cytoplasmic domain, and a luminal C-terminus [35]. CLN6 contains no asparagine-linked glycosylation sites and can form dimers upon overexpression [85]. Like other NCL membrane proteins (CLN3, CLN5, and CLN8), CLN6 has no homology with known proteins or functional domains, but the sequence is highly conserved across vertebrate species [83].

### CLN6 Function

The function of the CLN6 protein is unknown.

### Evolutionary Relationships- Alignment, Conserved Domains and Mutations

**Fig. (9) Conserved Domains of human CLN6.**
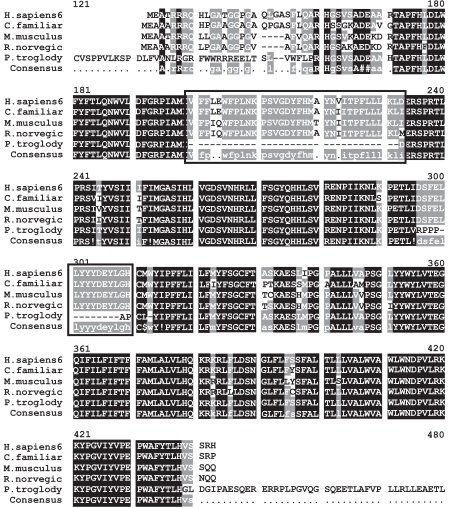
There are no known conserved domains in the human CLN6 protein. However, the MSA of CLN6 and its homologs show regions of high sequence similarity that may indicate potential unidentified domains. The MSA generated by MultAlin highlights individual residues with high consensus value (above 90%) in black and those with low consensus value (above 50%) in grey. All other neutral residues are not highlighted. There are regions of high sequence homology that are not indicated by Multalin; these are shown by black boxes around the conserved regions.

**Fig. (10) Phylogenetic tree of CLN6 homologs.**
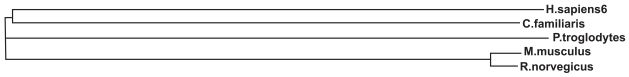
The phylogram tree is generated using the program ClustalW. In the above figure the branch lengths of the 5 homologous sequences are proportional to the amount of inferred evolutionary change.

The majority of vLINCL mutations result in a frameshift or nonsense change, with the introduction of a premature stop codon. However, a Portuguese patient was homozygous for a 3-bp deletion in exon 4 (c.460_462delATC) of *CLN6* which is predicted to remove a single amino acid (I154del) within the predicted third hydrophilic loop of the protein. This residue is within a region of the protein that is highly conserved across at least five species (human, mouse, cow, pig and chicken), suggesting that it is likely to have an important role in the function of CLN6. In addition, a Costa Rican patient was homozygous for an exon 4 missense mutation (c.368G→A) that changes glycine to aspartic acid (G123D) within the predicted third transmembrane domain. The introduction of a charged amino acid is predicted to disrupt this domain [83]. Sharp *et al*. (2003) [87] identified 8 mutations in *CLN6* bringing the total number of mutations found in this disorder to 18, of which 10 mutations affected single amino acids. These mutations are conserved across the vertebrate species in *C. familiaris, R. norvegicus, M. musculus* and *P. troglodytes* as noted in the multiple sequence alignment (Figs. **9** and **10**). 

## CLN7/MFSD8 (TURKISH VLINCL)

### Disease Progression

Children afflicted by the Turkish variant Late Infantile NCL show an onset of disease symptoms at a mean age of 5.1 years, ranging between 2 and 7 years. They suffer from the classical NCL disease symptoms including epileptic seizures, progressive psychomotor deterioration, visual failure, and premature death. However, the Turkish variant NCL patients suffer from seizures that are more severe than in patients that suffer from classical LINCL [88].

### Disease Autofluorescent Storage Material

Biochemical characteristics of AFSM in Turkish variant LINCL patients have not been determined.

### *CLN7* Gene and MFSD8 Protein

The Turkish variant LINCL gene, *CLN7*, was first characterized by Wheeler *et al*. [89] and was believed to be allelic to previously characterized NCL genes- *CLN8* [90, 91] and *CLN6* [92]. However, Siintola *et al*. [93] mapped *CLN7* to a unique locus on chromosome 4q28.1-q28.2 using a genomewide scan with Single Nucleotide Polymorphism (SNP) markers and homozygosity mapping. *CLN7* or the *MFSD8* gene belongs to the major facilitator superfamily of transporter proteins and encodes MFSD8, a putative lysosomal transporter.

### MFSD8 Structure 

MFSD8 (Major Facilitator Superfamily Domain-containing protein-8) is predicted to be a 518 amino acid protein that localizes mainly to the lysosomal compartment. It is approximately 58 kD in size with 12 predicted transmembrane domains. MFSD8 is expressed ubiquitously with several alternative splice variants that were detected by Northern blot and Expressed Sequence Tag (EST) database analysis [93].

### MFSD8 Function

The function of MFSD8 is unknown.

### Evolutionary Relationships- Alignment, Conserved Domains and Mutations

**Fig. (11) Conserved Domains of human CLN7.**
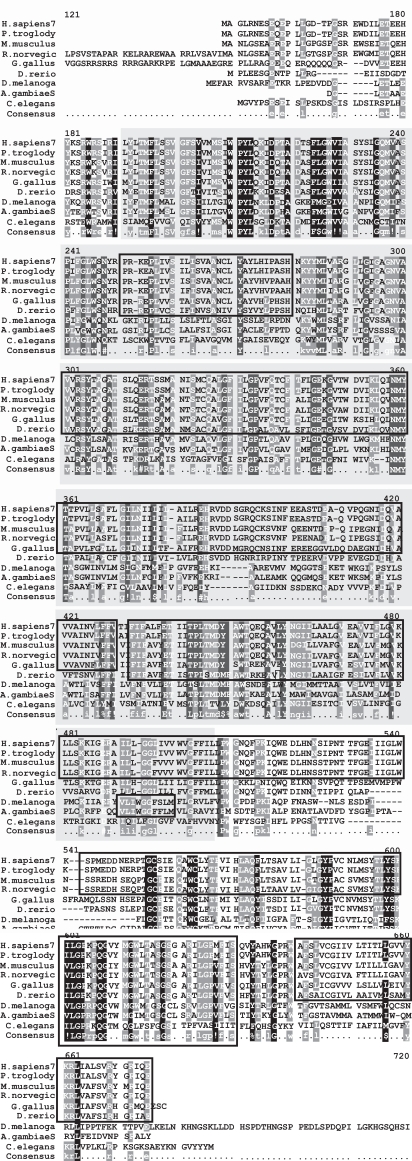
The conserved domain of human CLN7 is Major Facilitator Superfamily. (pfam07690) indicated by the box shaded in gray that spans the multiple sequence alignment (MSA). It is 346 amino acids in length and aligns 82.9% with the human PPT1 protein from residue 42 to residue 354. The MSA generated by MultAlin highlights individual residues with high consensus value (above 90%) in black and those with low consensus value (above 50%) in grey. All other neutral residues are not highlighted. There are regions of high sequence homology that are not indicated by Multalin; these are shown by black boxes around the conserved regions.

**Fig. (12) Phylogenetic tree of CLN7 homologs.**
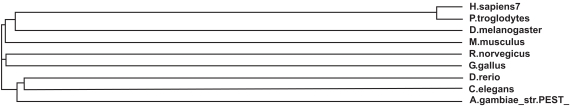
The phylogram tree is generated using the program ClustalW. In the above figure the branch lengths of the 9 homologous sequences are proportional to the amount of inferred evolutionary change.

Analysis of the MFSD8 amino acid sequence using the Pfam (Protein FAMily) domain database revealed that MFSD8 contains a Major Facilitator Superfamily (MFS) domain and a sugar transporter domain between amino acid positions 42 - 477 and 72 - 147 respectively. A BLAST search of MFSD8 returned several homologs for MFSD8 in different species- *H. sapiens, P. troglodytes, M. musculus, R. norvegicus, G. gallus, D. melanogaster, D. rerio, A. gambiae* and *C. elegans*, suggesting that it is an evolutionarily conserved protein (Figs. **11** and **12**).

Of the six mutations that Siintola *et al*. (2007) [93] identified in the *MFSD8* gene two of them- GLY310ASP and GLY429ASP resulted in amino acid substitutions in exon 10 and 12 respectively, in the Turkish patients carrying the defective *MFSD8* gene. A third mutation, TYR298TER, was identified in an Indian patient. This results from a transversion event in exon 10 of the *MFSD8* gene which gives rise to a truncated protein.

## CLN8 (EPMR/NORTHERN EPILEPSY)

### Disease Progression

Northern epilepsy, also known as progressive epilepsy with mental retardation (EPMR), is caused by a Finnish founder mutation in the CLN8 gene. It has the most protracted course of all the NCLs and is characterized by the onset of generalized seizures between 5 and 10 years of age and subsequent progressive mental retardation. Visual problems are not severe, myoclonus does not exist and the clinical progression of the disease is much slower [71, 91].

### Disease Autofluorescent Storage Material

Northern epilepsy, like Finnish vLINCL is pathologically characterized by intraneuronal cytoplasmic deposits of autofluorescent granules. Mitochondrial ATP synthase subunit c is the main stored protein in both disorders [71].

### *CLN8* Gene and CLN8 Protein

*CLN8* encodes a ubiquitously expressed 286 amino acid transmembrane protein [94]. It has been suggested that CLN8 is an endoplasmic reticulum (ER) resident protein that recycles between the ER and ER–Golgi intermediate compartment (ERGIC) in non-neuronal cells and the ER in neuronal cells [95].

### CLN8 Structure

**Fig. (13) Conserved Domains of human CLN8.**
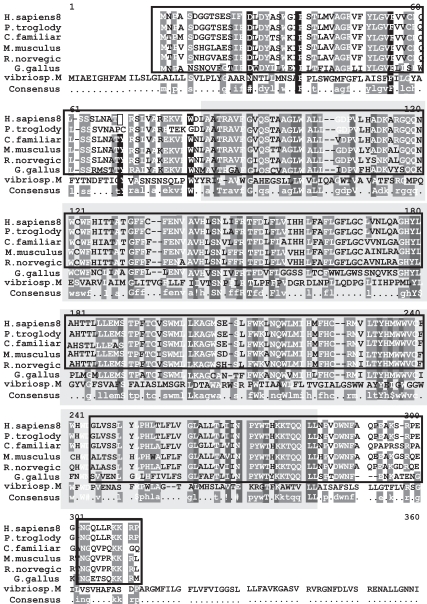
The conserved domain of human CLN8 shows a high degree of conservation with the TLC, TRAM, LAG1 and CLN8 homology domains (smart00724). These protein domains contain at least 5 transmembrane alpha-helices. Lag1p and Lac1p are essential for acyl-CoA-dependent ceramide synthesis, TRAM is a subunit of the translocon and the CLN8 gene is mutated in Northern epilepsy syndrome. The family may possess multiple functions such as lipid trafficking, metabolism, or sensing. Trh homologues possess additional homeobox domains. The conserved domain is indicated by the box shaded in gray that spans the multiple sequence alignment (MSA). It is 208 amino acids in length and aligns 95.7% with the human CLN8 protein from residue 67 to residue 258. The MSA generated by MultAlin highlights individual residues with high consensus value (above 90%) in black and those with low consensus value (above 50%) in grey. All other neutral residues are not highlighted. There are regions of high sequence homology that are not indicated by Multalin; these are shown by black boxes around the conserved regions.

CLN8 is a 33kD non-glycosylated transmembrane protein. It contains an ER-retrieval signal KKRP in the C-terminus (aa 283-286) [95]. Sequence homology links CLN8 to a large eukaryotic protein family of TLC-domain homologs (TRAM, Lag1, CLN8 homology domain; SMART accession number SM00724), (Fig. **13**) [96]. Members of this family have been shown to facilitate translocation of nascent polypeptide chains into the ER and export of glycosylphosphatidylinositol-anchored proteins out of the ER [35, 97-99].

### CLN8 Function

The function of CLN8 protein is unknown.

### Evolutionary Relationships- Alignment, Conserved Domains and Mutations

**Fig. (14) Phylogenetic tree of CLN8 homologs.**
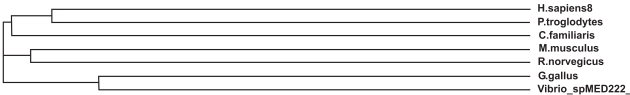
The phylogram tree is generated using the program ClustalW. In the above figure the branch lengths of the 7 homologous sequences are proportional to the amount of inferred evolutionary change.

Northern Epilepsy patients have been observed to carry some of the following mutations, ARG24GLY, TRP263-CYS, ARG204CYS, 1-BP DEL, 88G, ALA30PRO, and 1-BP DEL, 66G. All these residues are highly conserved in all vertebrate species that have homologs of CLN8-*H. sapiens, P. troglodytes, C. familiaris, M. musculus, R. norvegicus, G. gallus* and *Vibrio* sp. MED222 (Figs. **13** and **14**).

## CLN10/CATHEPSIN D (CONGENITAL NCL)

### Disease Progression

Congenital NCL (CNCL) is a rare congenital disorder that was first described in 1941 [100]. It is characterized by microencephaly, rigidity, seizures, and respiratory difficulties resulting in death usually within a few hours or weeks after birth. This disorder has been identified in about ten individuals who upon post-mortem examination reveal a small, firm brain with severe neuronal loss, gliosis, white matter lacking myelin and accumulation of storage material within cells in the brain and the reticuloendothelial system [100-105].

### Disease Autofluorescent Storage Material

Sintolla *et al*. [105] showed that the storage material typical of CNCL affected individuals stained positively for the sphingolipid activator protein D which is also found only in INCL patients.

### *CLN 10/ CTSD* Gene and Cathepsin D Protein

*CTSD* gene consists of 9 exons and is located on chromosome 11p15.5 [106-108]. It encodes a 412 amino acid protein cathepsin D (CTSD) which is a lysosomal aspartic protease that belongs to the pepsin family [109, 110].

### Cathepsin D Structure

Mature cathepsin D is a two-chain, glycosylated, lysosomal aspartic protease [111].

It is classified in the A1 family of aspartyl proteinases. Cathepsin D consists of two polypeptides encoded by the *CTSD* gene. The *CTSD* gene first encodes a preproenzyme which undergoes several proteolytic processing steps resulting in a single-chain active polypeptide that is 43kD in size. This active single chain polypeptide is further processed resulting in a mature form consisting of two polypeptides [109] interlinked by disulphide bridges [112]. Both the polypeptides contain an aspartic acid residue essential for the enzymatic activity of the mature protein [109, 113]. The mature protease is 31kD in size.

### Cathepsin D Function

Cathepsin D is a ubiquitously expressed lysosomal aspartic protease that belongs to the pepsin family [110]. There are several proteins described to function as substrates of CTSD *in vitro* but the *in vivo* substrates are still unknown [35]. Aspartyl proteinases consist of two domains each of which contains an aspartate residue. The residues come together and link to a water molecule at the active site where the substrate peptide bond is hydrolyzed. Hence, mutation of the aspartate residues which may be present at distant regions of the protein, results in elimination of enzymatic activity of cathepsin D without affecting its processing [114, 115].

### Evolutionary Relationships- Alignment, Conserved Domains and Mutations

**Fig. (15) Conserved Domains of human CLN10.**
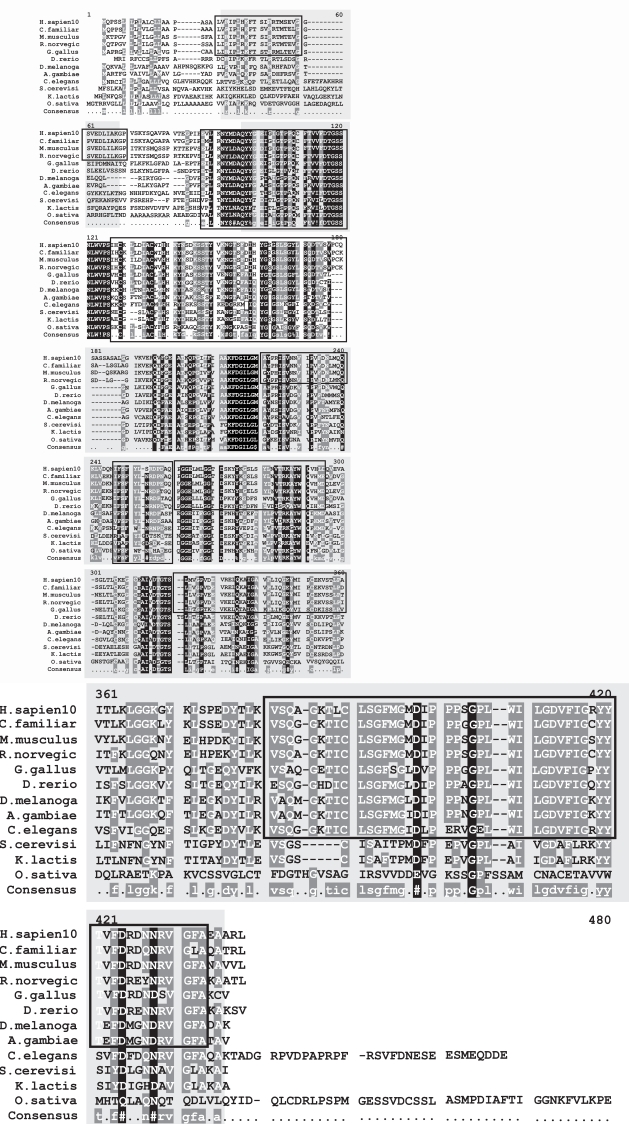
The conserved domains of human CLN10 i.e., the Cathepsin D protein are A1 Propeptide (pfam07966) and the Eukaryotic aspartyl protease (pfam00026) indicated by the boxes shaded in gray that span the multiple sequence alignment (MSA) from the 5’ to the 3’ end respectively. The A1 Propeptide domain is 29 amino acids in length and aligns 96.6% with the human CLN10 protein from residue 22 to residue 49. The Eukaryotic aspartyl protease domain is 314 amino acids in length and aligns 100% with the human CLN10 protein from residue 78 to residue 409. The MSA generated by MultAlin highlights individual residues with high consensus value (above 90%) in black and those with low consensus value (above 50%) in grey. All other neutral residues are not highlighted. There are regions of high sequence homology that are not indicated by Multalin; these are shown by black boxes around the conserved regions.

**Fig. (16) Phylogenetic tree of CLN10 homologs.**
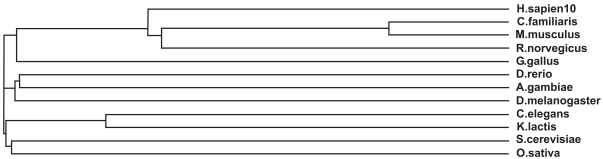
The phylogram tree is generated using the program ClustalW. In the above figure the branch lengths of the 12 homologous sequences are proportional to the amount of inferred evolutionary change.

Cathepsin D is a conserved protein through evolution and has 15 homologous gene sequences present in H. sapiens, C. familiaris, M. musculus, R. norvegicus, G. gallus, D. rerio, D. melanogaster, A. gambiae, C. elegans, S. cerevisiae, K. lactis, M. grisea, N. crassa, A. thaliana and O. sativa (Figs. **15** and **16**). Though the aspartyl protease domain (pfam00026) is conserved in all of the homologs, the A1 propeptide (pfam07966) is present only in humans and fruitflies. Cathepsin D mutant flies exhibit several features related to NCL pathology such as the progressive accumulation of AFSM in neurons and modest neurodegeneration [116]. It is interesting to see that A. thaliana and O. sativa have SapB_2 (pfam03489, Saposin-like type B, region 2) and SapB_1 (pfam05184, Saposin-like type B, region 1) conserved domains present in their homologous cathepsin D protein sequence as saposin D is a component of the ultrastructural AFSM that accumulates in CNCL patients. Sheep [117] and mice [118] deficient in functional cathepsin D protein also recapitulate CNCL phenotypes and are being exploited as model organisms to study the disease. Some of the important mutations occurring in patients that results in CNCL are PHE229ILE, TRP383CYS [119] and Y255X [105].

## DISCUSSION

Eight NCL genes have been identified- *CLN1*, *CLN2*, *CLN3*, *CLN5*, *CLN6*, *CLN7*, *CLN8* and *CLN10*. Their gene products- PPT1, TPP1, CLN3, CLN5, CLN6, MFSD8, CLN8 and cathepsin D, respectively, are highly conserved. The high degree of sequence similarity between the homologs implies a conserved functional role to each of the corresponding human NCL proteins. Consequently, several model organisms have been used to study the various clinical and pathological features of the NCL genes. However, despite extensive research the functional role of several NCL proteins remains elusive. 

The NCLs are neurodegenerative lysosomal storage disorders that show pathological accumulation of autofluorescent storage material in the lysosomes of the tissues of the affected individuals. However, beyond the cursory grouping of these proteins resulting in a disease with similar clinical manifestations, there is not sufficient sequence or domain similarity between the NCL genes to assign a similar function to them or to group them in the same biological pathway.

We have therefore performed a comparative biological analysis of the NCL proteins in the context of phylogeny. A sound classification of gene family relationships is a prerequisite in understanding how genes evolved along with the proteins that they encode. A reliable gene phylogeny is a powerful tool with which the structure and function of uncharacterized proteins can be predicted and mechanisms by which new genes appeared and assumed characteristic functions can be inferred. Such a phylogenic reconstruction also allows a better understanding of how biochemical pathways were established and what the role of the homologous proteins was in their evolutionary ancestors. Therefore, co evolutionary relationships can be analyzed in a meaningful way to elucidate the dynamics among proteins and to understand the links between genomic change and how this change is manifested morphologically [120, 121].

**Fig. (17) Clade Confines of the various NCL proteins.**
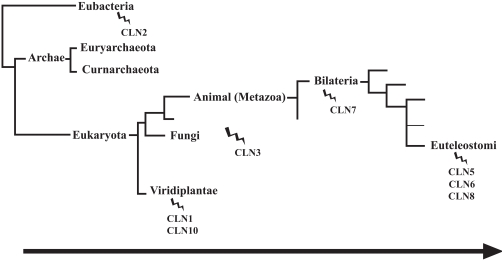
CLN2 is conserved in the Eubacteria clade. CLN1 and CLN10 are conserved in the Viridiplantae group. CLN3 appears in the Fungi and Metazoan group. CLN7 is conserved in Bilateria. CLN5, CLN6 and CLN8 are conserved in the Euteleostomi group.

The generation of multiple sequence alignments and corresponding phylogenetic trees of each of the NCL proteins is the first step in attempting to resolve the evolutionary origins of these individual proteins. A look at when the NCL proteins first appeared in the tree of life allows a grouping of them based on their corresponding cladistic confines. CLN2 appears the earliest in the Bacterial clade in the organisms *Pseudomonas* sp. 101 and *Xanthomonas* sp. T-22. TPP1 has significant sequence similarities to two previously characterizedbacterial pepstatin-insensitive carboxyl peptidases from *Xanthomonas* and *Pseudomonas*. CLN2 is synthesized as an inactive zymogen that is autocatalytically converted to an active serine protease at acidic pH in the lysosome [37]. BLAST searches were also able to identify a sequence with homology to CLN2 in *Sulfolobus solfataricus* which is present in the Archae clade (Figs. **3**, **4** and **17**). 

Next, CLN1 and CLN10 appear in the eukaryotic clade, Viridiplantae. Their resulting NCL pathology shares the same predominant AFSM of saposins. CLN1 and CLN10 also encode soluble lysosomal enzymes whose functions are known. CLN1 and CLN10 protein sequences contain the conserved domains for palmitoyl protein thioesterase and eukaryotic aspartyl protease, respectively, which span the entire length of the protein. A common role for PPT1 and cathepsin D appears to be in breakdown of lysosomal substrates, either through lipid removal from protein or degradation of proteins, respectively.

CLN3 appears in the Fungal/ Metazoan clade. In LINCL and JNCL the primary component of the AFSM is the mitochondrial ATP synthase subunit C. The function of CLN3 is unknown though it is a very well conserved protein that is known to localize to the lysosomal membrane. 

CLN7 or MFSD8 appears in the Bilateria clade of the metazoan organisms that include the nematode worms, *Caenorhabditis elegans*. *C. elegans* are the only homolog of CLN7 that also contain the Major Facilitator Superfamily domain found in humans. Lastly, CLN5, 6 and 8 appear in the more recent Euteliostomi clade in the organism *Danio rerio* also known as Zebra fish. In addition to its eukaryotic homologs identified by HomoloGene, BLAST searches identify a bacterial species protein in *Vibrio* sp. MED222 that shares sequence similarity with CLN8. CLN5 and CLN6 multiple sequence alignments indicate that there is a high degree of sequence conservation among the homologs however; no conserved domains have been identified. CLN8 contains the conserved domain TRAM which contains at least five transmembrane alpha helices. TRAM containing proteins may possess multiple functions such as lipid trafficking, metabolism, or sensing. However, the precise function of CLN8, like CLN5, CLN6 and CLN7 is unknown. CLN5 is the only soluble lysosomal enzyme among these more recently evolved proteins. The remaining are all transmembrane proteins occurring in various organelles in the endosomal/ lysosomal pathway. It is possible that CLN1 and CLN10 as well as CLN5, CLN6 and CLN8 that are subsequently confined to the same clade may have evolved along with common substrates or other proteins in related pathways. Thus, the classification of NCL proteins based on their phylogenetic relationships with each other provides an alternative way to group the NCLs. This grouping does however appear to overlap somewhat with the types of AFSM associated to the loss of the NCL-protein. Thus, continued studies on how the NCL proteins and proteins with biological relevance to NCL-associated pathways evolved may provide clues about the function of NCL-proteins.
